# Hemostatic and Inflammatory Biomarkers are Associated with Functional Limitations after Venous Thromboembolism: A Prospective Cohort Study

**DOI:** 10.1055/a-2574-8775

**Published:** 2025-04-25

**Authors:** Daniel Steiner, Stephan Nopp, Timothy Hoberstorfer, Oliver Schlager, Ingrid Pabinger, Benedikt Weber, Cihan Ay

**Affiliations:** 1 Division of Hematology and Hemostaseology, Department of Medicine I, Medical University of Vienna, Vienna, Austria; 2 Division of Angiology, Department of Medicine II, Medical University of Vienna, Vienna, Austria; 3 Department of Dermatology, Medical University of Vienna, Vienna, Austria

**Keywords:** biomarkers, deep vein thrombosis, functional status, pulmonary embolism, venous thromboembolism

## Abstract

Functional limitations often persist in patients with venous thromboembolism (VTE). The relevance of biomarkers for these outcomes remains unexplored. Therefore, we aimed to investigate the association of hemostatic, inflammatory, and cardiovascular biomarkers with functional limitations 3 months after VTE. We conducted a prospective cohort study, including patients with acute VTE within 21 days of diagnosis. Biomarker levels (D-dimer, fibrinogen, factor VIII [FVIII], von Willebrand factor antigen [VWF], C-reactive protein [CRP], troponin T, N-terminal pro-B-type natriuretic peptide [proBNP]) were measured at inclusion and 3 months. Functional limitations at 3 months were evaluated with the post-VTE functional status (PVFS) scale (0–4, higher indicating more limitations). The association of biomarkers with functional limitations was assessed with proportional odds models adjusted for confounders. Furthermore, we evaluated the area under the receiver operating characteristic curve (AUC-ROC) for the presence of slight-to-severe functional limitations. Overall, we included 290 patients (41.4% of women) with a median age of 54.9 years (interquartile range [IQR]: 43.1–64.2). D-dimer, fibrinogen, FVIII, VWF, and CRP measured at inclusion were independently associated with functional limitations at 3 months. VWF showed the most favorable AUC-ROC (0.62, 95% CI, 0.55–0.69). In patients with pulmonary embolism, troponin T and proBNP were not associated with functional limitations. At the 3-month follow-up, D-dimer was the only biomarker independently associated with functional limitations, yielding an area under the curve (AUC) of 0.62 (95% CI, 0.55–0.69). In conclusion, we identified biomarkers independently associated with functional limitations 3 months after VTE. Our results indicate a role of these biomarkers in the early identification of patients at risk of persistent functional limitations and suggest their involvement in the underlying mechanisms.

Venous thromboembolism (VTE), which encompasses pulmonary embolism (PE) and deep vein thrombosis (DVT), is a common cardiovascular disease with an annual incidence ranging from 1 to 2 per 1,000 individuals. 1 It poses a major burden on the population, considering that VTE associated with hospitalization is one of the leading causes of disability-adjusted life-years lost. 2 Furthermore, VTE has a considerable financial impact on health care systems, with total estimated costs within the European Union-28 ranging from €1.5 to €13.2 billion per year. 3 Several strategies to reduce the burden of VTE have been proposed, such as increasing VTE awareness, improving VTE risk assessment, providing appropriate use of thromboprophylaxis, and ensuring accurate VTE surveillance. 4

There are several more implications arising from VTE for affected patients. Apart from traditional clinical outcomes such as recurrence, anticoagulation-associated bleeding, and mortality, patients experiencing a VTE event are at risk of impaired quality of life, physical incapacity, and psychosocial distress in the short- and long-term periods. 5 6 7 8 9 10 11 12 13 14 15 16 17 18 19 20 21 22 23 To optimally assess these patient-centered outcomes, a standard set of outcome measures has been developed recently. 24 Among those measures, the post-VTE functional status (PVFS) scale has been selected to evaluate functional limitations after VTE. The PVFS scale is intended to capture the whole range of functional limitations in patients with VTE, including patients with both PE and DVT. 25 26 27 When applying the PVFS scale in a prospective cohort study of patients with acute VTE, we observed an overall improvement in functional limitations during a 12-month follow-up. 28 However, patients did not return to pre-VTE functional status despite standard anticoagulation treatment. Although we were able to identify several clinical characteristics associated with functional limitations, a considerable variability in data could not be explained by clinical characteristics alone. 28 This unexplained persistence of functional limitations might suggest that the acute VTE event initiates an underlying process, which results in physical impairment. However, further details of this process and the role of biomarkers have not been explored.

Therefore, we aimed to investigate the association of biomarkers, focusing on parameters reflecting the hemostatic, inflammatory, and cardiovascular systems, with persisting functional limitations in patients with VTE.

## Materials and Methods

### Study Design and Patient Population

This project was conducted within the framework of the ongoing BACH-VTE study—“A prospective observational study to investigate predictors of Bleeding and Assess long-term outComes on Health in patients with Venous ThromboEmbolism.” The BACH-VTE study is a prospective, observational cohort study at the Medical University of Vienna, Austria. The detailed study characteristics, including schedule, inclusion and exclusion criteria, and outcomes, have been published previously. 27 28 29 30 Shortly, adult patients with an objectively confirmed acute DVT and/or PE are eligible for inclusion within 21 days of diagnosis. The main exclusion criterion is therapeutic anticoagulation for any reason in the 3 months before DVT/PE diagnosis. Outcomes of the BACH-VTE study include incidence and risk factors of bleeding, functional limitations, postthrombotic and post-PE syndromes, quality of life, and psychological sequelae, among others. The follow-up visits are incorporated into routine clinical care, with the first follow-up at 3 months and the second at 1 year. The last follow-up is scheduled at 5 years.

For the present analysis, we excluded patients with active cancer, pregnancy, and puerperium at the time of diagnosis. Further, we only considered patients included between July 2020 and November 2023 who completed the first follow-up visit. We assessed clinical characteristics and demographics in a face-to-face interview at study inclusion and checked self-reported data with medical records. Furthermore, we performed a blood draw by sterile venipuncture. After 3 months, we performed the follow-up visit, including an assessment of functional limitations and another blood draw.

All included patients provided written informed consent prior to study inclusion. The study was conducted according to the principles of the Declaration of Helsinki and was approved by the local Ethics Committee of the Medical University of Vienna (EK 1045/2020). Study data were collected and managed using REDCap electronic data capture tools hosted at the Medical University of Vienna. 31

### Assessment of Functional Limitations

We assessed functional limitations with the PVFS scale, which is an ordinal measure modeled after the modified Rankin scale for patients with stroke and intends to capture the whole range of functional limitations after VTE. 25 26 27 The scale ranges from 0 to 4, with the following distinct grades: 0, no functional limitations; 1, negligible functional limitations; 2, slight functional limitations; 3, moderate functional limitations; and 4, severe functional limitations. Thus, higher scale grades indicate more functional limitations. In our study, we assessed the PVFS scale through a structured interview at the first follow-up, that is, 3 months after diagnosis, and asked patients about their functional limitations prior to the VTE diagnosis as suggested in the scale manual. 26

### Biomarker Measurement

We intended to evaluate the association of functional limitations with readily available biomarkers reflecting the hemostatic, inflammatory, and cardiovascular systems. Thus, we considered the following parameters: D-dimer, fibrinogen, factor VIII (FVIII), von Willebrand factor antigen (VWF), and C-reactive protein (CRP). Furthermore, we evaluated troponin T and N-terminal pro-B-type natriuretic peptide (proBNP) in patients with PE. All biomarkers were routinely measured at study inclusion and the first follow-up visit at 3 months. However, for some hemostatic biomarkers, that is, FVIII and VWF, this routine measurement was implemented during the conduct of the study rather than from the beginning. Therefore, missing value rates for these biomarkers, especially at the time of study inclusion, are higher than for other biomarkers. Units, normal ranges, and other specifications of biomarkers are summarized in Supplementary Table S1 (available in the online version only).

We defined two distinct aims for our study. First, we aimed to investigate the association of biomarkers measured at study inclusion—within 21 days from VTE diagnosis—and functional limitations after 3 months. This analysis assesses the prognostic value of these biomarkers, potentially allowing for the early identification of patients at risk for persisting functional limitations. Second, we examined the association between biomarkers measured at follow-up—3 months after VTE diagnosis—and functional limitations at this time point. This analysis reflects the diagnostic value of biomarkers for persisting functional limitations and might, therefore, generate hypotheses regarding the underlying pathophysiological mechanisms of long-term limitations.

### Statistical Analysis

Categorical variables are summarized as absolute and relative frequencies, and continuous variables as median and 25th and 75th percentiles, that is, interquartile range (IQR). Missing values are reported but were not imputed. The correlation between all considered biomarkers was evaluated with Spearman's rank correlation coefficient and displayed graphically. To investigate the association between biomarkers and functional limitations at follow-up, we built proportional odds logistic regression models (cumulative logit link models). The dependent variable was the PVFS scale grade 3 months after the VTE event, the independent variables were the biomarkers of interest. Every biomarker was evaluated in a separate model, and we considered levels at study inclusion, that is, within 21 days of VTE diagnosis, and at follow-up, that is, 3 months after diagnosis. Due to right-skewed distribution, we log-transformed D-dimer, CRP, troponin T, and proBNP. First, we performed univariable modelling, resulting in unadjusted odds ratios (ORs) and corresponding 95% confidence intervals (CIs). Then, we adjusted for clinical characteristics for which we previously identified an association with functional limitations and for potential confounders, that is, sex, age, body mass index (BMI), VTE type (i.e., PE or DVT), and history of cardiovascular or pulmonary disease. 28 Age was modeled in a nonlinear fashion, using restricted cubic splines with 3 degrees of freedom. The knots were placed at the 5th, 35th, 65th, and 95th percentiles. To account for functional limitations unrelated to VTE, we adjusted for PVFS scale grade before VTE diagnosis. The resulting unadjusted and adjusted ORs indicate the likelihood of having a higher PVFS scale grade in relation to the respective increase in each biomarker.

To investigate the ability of the biomarkers to distinguish patients with functional limitations from those without, we compared proportions of patients per PVFS scale categories in patients with high (≥50th percentile) and low (<50th percentile) biomarker levels. Furthermore, we evaluated the discriminative performance of biomarkers for the presence of PVFS scale >1 with the area under the receiver operating characteristic curve (AUC-ROC). Corresponding CIs were computed with 2,000 stratified bootstrap replicates.

All analyses were done in R version 4.3.3 using the rms package. 32 33

## Results

Our study cohort encompassed 290 patients with a median (IQR) age of 54.9 (43.1–64.2) years, including 41.4% women and 134 (46.2%) patients with PE. Detailed clinical characteristics are displayed in Table 1 . The median (IQR) PVFS scale grade at 3 months was 1 (0–2).

**Table 1:** Patient demographics and clinical characteristics

	Full cohort ( *n* = 290)
Female, *n* (%)	120 (41.4)
Age, median (IQR)	54.9 (43.1–64.2)
BMI, median (IQR) a	27.7 (24.7–31.6)
Type of VTE, *n* (%)	–
Pulmonary embolism b	134 (46.2)
Deep vein thrombosis	156 (53.8)
Unprovoked VTE, *n* (%)	174 (60.0)
Provoked VTE, *n* (%) c	116 (40.0)
Major persisting risk factor	25 (8.6)
Major transient risk factor	38 (13.1)
Minor transient risk factor	66 (22.8)
History of VTE, *n* (%)	85 (29.3)
History of cardiovascular or pulmonary disease, *n* (%) d	67 (23.1)
Smoking, *n* (%)	–
Current	71 (24.5)
Former	63 (21.7)
Never	156 (53.8)

Abbreviations: BMI, body mass index; IQR, interquartile range; VTE, venous thromboembolism.

a Data missing for one patient.

b With or without deep vein thrombosis.

c Some patients had more than one risk factor; patients with cancer, patients with pregnancy, and patients in the postpartum period were excluded.

d Including coronary artery disease, chronic heart failure, arrhythmia, peripheral artery disease, cerebrovascular disease, and chronic pulmonary disease.

### Biomarkers at Study Inclusion

The median (IQR) time from VTE diagnosis to study inclusion and blood draw was 3 (1–7) days. Median (IQR) biomarker levels at study inclusion are shown in Supplementary Fig. S1 (available in the online version only), and a correlation matrix between all considered biomarkers is shown in Supplementary Fig. S2 (available in the online version only). D-dimer was missing in 20 patients, fibrinogen in 15, FVIII in 75, VWF in 62, and CRP in 15. Detailed clinical characteristics of patients with and without missing biomarker values are shown in Supplementary Table S2 (available in the online version only). In 134 patients with PE, troponin T was missing in 24 and proBNP in 23 patients.

**Fig. 1:**
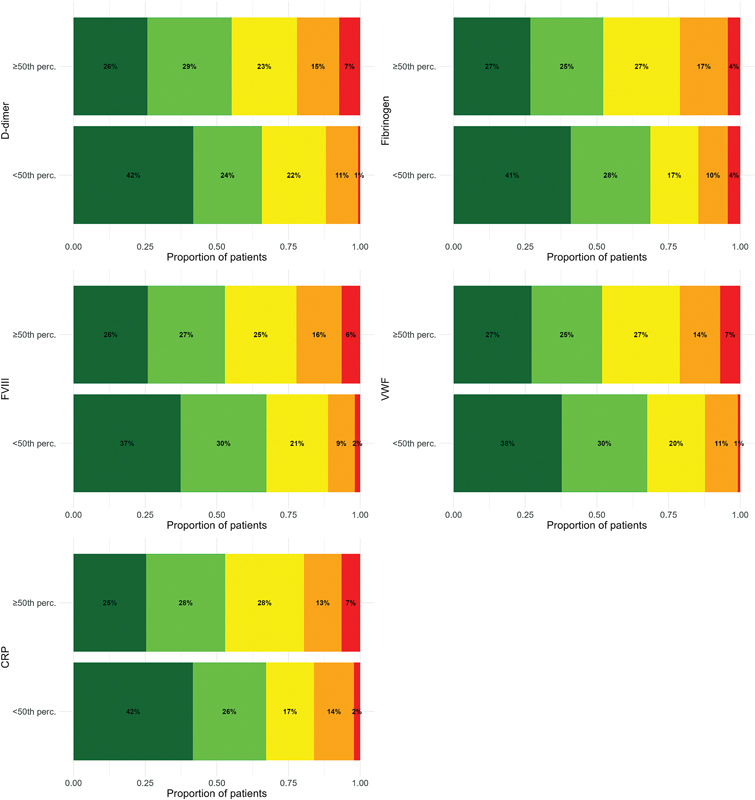
Proportions of patients per post-VTE functional status (PVFS) scale category at 3 months with high (≥50th percentile) and low (<50th percentile) biomarker levels measured at study inclusion. Dark green refers to PVFS scale of 0 (no functional limitations), light green to 1 (negligible functional limitations), yellow to 2 (slight functional limitations), orange to 3 (moderate functional limitations), and red to 4 (severe functional limitations). CRP, C-reactive protein; FVIII, factor VIII; perc, percentile; VTE, venous thromboembolism; VWF, von Willebrand factor antigen.

All hemostatic and inflammatory biomarkers measured at study inclusion showed a significant and independent association with functional limitations after 3 months ( Table 2 ). A 50-unit increase in FVIII and VWF was associated with 1.21-fold increased odds of having a higher PVFS scale grade after 3 months (OR: 1.21, 95% CI: 1.01–1.45 for both). In patients with PE, cardiac biomarkers were not significantly associated with functional limitations. When stratifying patients by biomarker levels into a high-level and a low-level group (≥50th percentile and <50th percentile, respectively), hemostatic and inflammatory biomarkers measured at study inclusion showed a moderate performance in differentiating patients with high and low PVFS scale grades ( Fig. 1 ). In patients with PE, cardiac biomarkers showed a poor to moderate performance in differentiating patients ( Supplementary Fig. S3 [available in the online version only]). The absolute and relative frequencies of patients within the PVFS scale grade according to biomarker levels are shown in Supplementary Table S3 (available in the online version only). Less than 1% of patients with a study inclusion D-dimer or VWF level below the cohort median had a PVFS scale grade of 4, and nearly 70% of those with a study inclusion fibrinogen level below the cohort median had a PVFS scale grade of 0 or 1. The discriminatory performance of hemostatic and inflammatory biomarkers for the presence of PVFS scale grade >1 was moderate, with AUC-ROCs (95% CIs) ranging from 0.57 (0.51–0.64) for CRP to 0.62 (0.55–0.69) for VWF ( Supplementary Table S4 [available in the online version only]).

**Table 2:** Association between biomarker levels at study inclusion and functional limitations at 3 months

	Unadjusted OR (95% CI)	Adjusted OR (95% CI) a
D-dimer (per double, μg/mL)	1.20 (1.05–1.37)	1.17 (1.01–1.35)
Fibrinogen (per 50 mg/dL increase)	1.11 (1.02–1.22)	1.09 (1.00–1.20)
FVIII (per 50% increase b )	1.27 (1.07–1.50)	1.21 (1.01–1.45)
VWF (per 50% increase b )	1.34 (1.13–1.59)	1.21 (1.01–1.45)
CRP (per double, mg/dL)	1.12 (1.02–1.23)	1.11 (1.00–1.23)
Troponin T c (per double, ng/L)	1.16 (0.93–1.44)	1.10 (0.84–1.46)
proBNP c (per double, pg/mL)	1.13 (0.97–1.31)	1.05 (0.87–1.27)

Abbreviations: CI, confidence interval; CRP, C-reactive protein; FVIII, factor VIII; OR, odds ratio; proBNP, N-terminal pro-B-type natriuretic peptide; VWF, von Willebrand factor antigen.

a Adjusted for sex, age, body mass index, venous thromboembolism (VTE) type (i.e., pulmonary embolism or deep vein thrombosis), history of cardiovascular or pulmonary disease, and post-VTE functional status scale before VTE diagnosis. Age was modelled as a continuous variable using restricted cubic splines with four knots at the 5th, 35th, 65th, and 95th percentiles.

b Unit of FVIII and VWF is a percentage.

c Troponin T and proBNP were only evaluated in patients with pulmonary embolism, therefore, the multivariable model for these variables did not include VTE type.

### Biomarkers at 3 Months

The median (IQR) time from VTE diagnosis to follow-up blood draw was 98 (93–115) days. All biomarker values decreased compared to study inclusion ( Supplementary Fig. S4 [available in the online version only]). A correlation matrix of all considered biomarkers is shown in Supplementary Fig. S5 (available in the online version only). D-dimer was missing in 33 patients, fibrinogen in 28, FVIII in 39, VWF in 33, and CRP in 15. Detailed clinical characteristics stratified by missing biomarker levels are shown in Supplementary Table S5 (available in the online version only). In 134 patients with PE, troponin T was missing in 35 and proBNP in 30 patients.

**Fig. 2:**
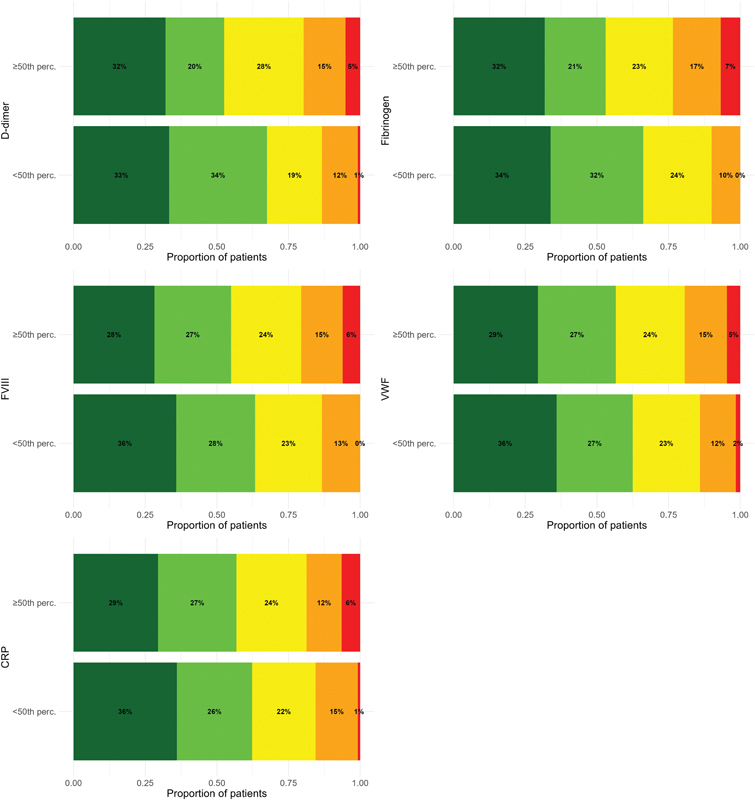
Proportions of patients per post-VTE functional status (PVFS) scale category at 3 months with high (≥50th percentile) and low (<50th percentile) biomarker levels measured at 3 months. Dark green refers to PVFS scale of 0 (no functional limitations), light green to 1 (negligible functional limitations), yellow to 2 (slight functional limitations), orange to 3 (moderate functional limitations), and red to 4 (severe functional limitations). FVIII, factor VIII; CRP, C-reactive protein; perc, percentile; VTE, venous thromboembolism; VWF, von Willebrand factor antigen.

After adjusting for confounders, only D-dimer remained significantly associated with functional limitations (adjusted OR [95% CI], 1.22 [1.00–1.49]; Table 3 ). Similar to biomarkers measured at study inclusion, biomarkers measured after 3 months showed a moderate performance in differentiating patients with high and low PVFS scale grades ( Fig. 2 ). In patients with PE, cardiac biomarkers showed a poor performance ( Supplementary Fig. S6 [available in the online version only]). The absolute and relative frequencies of patients within the PVFS scale grade according to biomarker levels measured at 3 months are shown in Supplementary Table S6 (available in the online version only). The discriminatory performance of biomarkers measured at 3 months for the presence of PVFS scale grade >1 was similar to the performance of biomarkers measured at study inclusion, with the highest AUC-ROC (95% CI) displayed by D-dimer and fibrinogen (0.62 [0.55–0.69] and 0.63 [0.55–0.69], respectively; Supplementary Table S7 [available in the online version only]).

**Table 3:** Association between biomarker levels at 3 months and functional limitations at 3 months

	Unadjusted OR (95% CI)	Adjusted OR (95% CI) a
D-dimer (per double, μg/mL)	1.35 (1.13–1.62)	1.22 (1.00–1.49)
Fibrinogen (per 50 mg/dL increase)	1.28 (1.12–1.48)	1.09 (0.92–1.29)
FVIII (per 50% increase b )	1.31 (1.07–1.60)	1.12 (0.90–1.39)
VWF (per 50% increase b )	1.28 (1.06–1.56)	1.06 (0.86–1.32)
CRP (per double, mg/dL)	1.19 (0.79–1.79)	1.04 (0.90–1.21)
Troponin T c (per double, ng/L)	1.16 (0.79–1.71)	1.31 (0.76–2.26)
proBNP c (per double, pg/mL)	1.21 (0.96–1.53)	0.95 (0.69–1.32)

Abbreviations: CI, confidence interval; CRP, C-reactive protein; FVIII, factor VIII; OR, odds ratio; proBNP, N-terminal pro-B-type natriuretic peptide; VWF, von Willebrand factor antigen.

a Adjusted for sex, age, body mass index, venous thromboembolism (VTE) type (i.e., pulmonary embolism or deep vein thrombosis), history of cardiovascular or pulmonary disease, and post-VTE functional status scale before VTE diagnosis. Age was modelled as a continuous variable using restricted cubic splines with four knots at the 5th, 35th, 65th, and 95th percentiles.

b Unit of FVIII and VWF is a percentage.

c Troponin T and proBNP were only evaluated in patients with pulmonary embolism, therefore, the multivariable model for these variables did not include VTE type.

## Discussion

In our prospective cohort study, we observed an independent association of hemostatic and inflammatory biomarkers at VTE diagnosis with functional limitations 3 months after VTE. Furthermore, higher D-dimer levels 3 months after VTE were significantly associated with persisting functional limitations.

The association between biomarkers and persisting functional limitations in patients with VTE has not been investigated previously. Since we considered biomarker measurements both at the time of diagnosis and after 3 months, we can draw several conclusions from our work. First, the independent association of biomarkers at the time of VTE diagnosis with functional limitations 3 months afterward underlines their prognostic value. Thereby, patients at a higher risk of functional limitations could be identified early in their disease process, enabling a comprehensive and close follow-up procedure with an early possibility of intervention, for example, with rehabilitation programs. The efficacy and cost-effectiveness of such interventions remain to be evaluated. Second, the independent association between D-dimer measured 3 months after diagnosis and functional limitations at the same time can be considered hypothesis-generating, potentially reflecting a mechanistic relationship. While details of a potential mechanism need to be evaluated in basic and translational research studies, the association may be explained by clot persistence, endothelial dysfunction or damage, a general state of hypercoagulability, or others.

The hemostatic and inflammatory biomarkers at hand have been investigated in various patient cohorts and clinical scenarios. High levels of VWF and FVIII have been associated with an increased risk of thrombosis. 34 35 At the same time, the release of VWF from Weibel Palade bodies can be triggered by cellular injury. 36 Thus, increased levels might be a sign of endothelial cell dysfunction, damage, or activation, presumably caused by VTE or at least more pronounced in patients with persisting functional limitations. 37 38 We observed a comparable association of VWF and FVIII with functional limitations. This is plausible due to their tight connection since VWF functions as a carrier for FVIII. 39 Apart from the general population, VWF and FVIII have been studied in patients with cancer, showing an association with a higher risk of thrombosis, poorer overall survival, and the presence of metastases. 40 41 42 Furthermore, FVIII has been associated with recurrent VTE and postthrombotic syndrome (PTS) in children and young adults. 43 Considering inflammatory biomarkers, a systematic review evaluated the association between biomarkers of inflammation and the development of PTS, suggesting an association of CRP and PTS. 44 This is in-line with the association between CRP and functional limitations observed in our study. Apart from CRP, intracellular adhesion molecule-1 showed promising results regarding the association with PTS, with statistical significance at several time points after proximal DVT and a dose–response association. 44 45 However, we cannot compare this result to our study since we only evaluated CRP. Overall, the association of hemostatic and inflammatory biomarkers measured at VTE diagnosis with functional limitations 3 months afterward complement the known prognostic role of these biomarkers for adverse outcomes in different patient cohorts. While we can only speculate on the details, possible mechanisms include thromboinflammation, endothelial cell damage or activation, hypercoagulability, or an increase in biomarkers as a surrogate for the extent and severity of the initial thrombotic event, reflecting an acute phase reaction.

When evaluating biomarkers 3 months after VTE, only D-dimer remained independently associated with functional limitations. Apart from its role in the diagnosis of VTE, the utility of D-dimer as a biomarker has been shown in several other clinical scenarios. Those include the prediction of VTE in ambulatory patients with solid cancer, the identification of patients at low risk of recurrent VTE, and the prediction of long-term risk of arterial and venous events, cancer, and mortality in patients with stable coronary heart disease. 46 47 48 Importantly, the measurement of D-dimer in these scenarios is performed when patients are not receiving anticoagulation, which is in contrast to our study. Regarding the long-term consequences of VTE, an association of D-dimer with recurrent VTE and PTS in children and young adults has been suggested. 43 Other explanations for an elevation in D-dimer values include the presence of cancer, advanced age, pregnancy, and puerperium, among others. 49 However, these factors are unlikely to explain the association with persisting functional limitations in our study due to the exclusion of patients with active cancer, pregnancy, and puerperium and the adjustment for important confounders, including age, sex, and comorbidities. D-dimer is a degradation product of fibrin. 50 Thus, higher levels might indicate the presence of residual thrombosis and/or increased hemostatic activity and hypercoagulability, which could explain the persistence of functional limitation. The hypercoagulability might be associated with endothelial cell function, platelet activation, inflammation, or others. Overall, this observation must be considered hypothesis-generating and should be investigated in more detail.

Surprisingly, we could not identify an association of troponin T and proBNP with persisting functional limitations in patients with PE. Troponins have been shown to be associated with short-term mortality and adverse outcomes in patients with PE. 51 A similar association has been observed for elevated BNP values. 52 Consequently, troponin levels have been incorporated into the risk assessment strategy of acute PE in current guidelines, and the role of proBNP to provide additional prognostic information has been acknowledged. 53 However, both troponins and proBNP have low specificity and positive predictive value while displaying high sensitivity and negative predictive value. 54 55 56 This could partially explain why we did not observe an association of elevated levels with persisting functional limitations. More importantly, the number of patients was very limited in this subgroup of our study, which most likely resulted in an underpowered analysis.

While we performed multivariable analyses per biomarker adjusted for potential confounders, we did not consider multiple biomarkers in a single model. The aim of this study was to generate hypotheses rather than predictive or causal models. Further, some biomarkers we considered certainly inherit collinearity due to their strong interplay, for example, VWF and FVIII, as can also be seen from the correlation matrices. Thus, incorporating all considered biomarkers in a single multivariable model and trying to interpret the respective ORs might be misleading and would not have added further value.

Our study has several limitations. First, the number of patients was limited, resulting in a considerable degree of uncertainty, especially in the subgroup analysis of patients with PE. Second, there was a considerable number of missing values in biomarker measurements, which is a result of the implementation of routine laboratory measurement during the conduct of the study rather than from the beginning. However, demographics and clinical characteristics were similar between patients with and without missing values. Third, our results must be considered hypothesis-generating as they neither represent a predictive nor a causal association between biomarkers and persisting functional limitations. Fourth, we only evaluated readily available biomarkers. Thus, future studies might take a broader approach and evaluate further biomarkers such as prothrombin fragment 1.2, thrombin–antithrombin complexes, or sP-selectin, among others. Lastly, the monocentric design of the study and the recruitment of patients in a tertiary care center might lead to a selection bias and limit the generalizability of the results.

## Conclusion

In conclusion, hemostatic and inflammatory biomarkers measured at VTE diagnosis and D-dimer measured after 3 months were independently associated with persisting functional limitations after VTE. This underlines their prognostic role for persisting functional limitations and suggests an underlying pathophysiological mechanism.
