# Supplementary material for: Hemostatic and Inflammatory Biomarkers are Associated with Functional Limitations after Venous Thromboembolism: A Prospective Cohort Study
Source: Semin Thromb Hemost. 2025 Apr 25;51(7):809–17. doi: 10.1055/a-2574-8775 (PMC12431823; doi:10.1055/a-2574-8775)
Supplement: Supplementary file 1 — Supplementary Material [file 10-1055-a-2574-8775-s250049oa.pdf]

**Supplementary Table S1** Detailed characteristics of biomarkers

| | Unit | Normal range[a] | Source | Method |
|---|---|---|---|---|
| D-dimer | µg/mL | <0.5 | Citrate tube | Quantitative assay, latex agglutination method |
| Fibrinogen | mg/dL | 200–400 | Citrate tube | Method according to Clauss |
| Factor VIII | % | 60–230 | Citrate tube | Photo-optical detection |
| von Willebrand factor antigen | % | 60–180 | Citrate tube | Latex agglutination method |
| C-reactive protein | mg/dL | <0.5 | Serum clot activator tube | Turbidimetry |
| Troponin T[b] | ng/L | 0–14 | Lithium-heparin plasma tube | Electrochemiluminescence immunoassay |
| N-terminal pro-B-type natriuretic peptide[a] | pg/mL | 0–125 | Lithium-heparin tube | Immunological method |

[a]According to the reference range of the local laboratory of the Medical University of Vienna, Austria.
[b]Troponin T and N-terminal pro-B-type natriuretic peptide were only assessed in patients with pulmonary embolism.

**Supplementary Table S2** Patient demographics and clinical characteristics of patients with and without missing biomarker values measured at study inclusion

| | No missing biomarker values ($n = 208$) | Missing biomarker values ($n = 82$) |
|---|---|---|
| Female, *n* (%) | 83 (39.9) | 37 (45.1) |
| Age, median (IQR) | 54.9 (42.7–64.4) | 54.4 (44.4–63.8) |
| BMI, median (IQR)[a] | 27.9 (24.7–32.0) | 27.0 (23.9–31.0) |
| Type of VTE, *n* (%) | – | – |
| Pulmonary embolism[b] | 99 (47.6) | 35 (42.7) |
| Deep vein thrombosis | 109 (52.4) | 47 (57.3) |
| Unprovoked VTE, *n* (%) | 125 (60.1) | 49 (59.8) |
| Provoked VTE, *n* (%)[c] | 83 (39.9) | 33 (40.2) |
| Major persisting risk factor | 21 (10.1) | 4 (4.9) |
| Major transient risk factor | 24 (11.5) | 14 (17.1) |
| Minor transient risk factor | 48 (23.1) | 18 (22.0) |
| History of VTE, *n* (%) | 58 (27.9) | 27 (32.9) |
| History of cardiovascular or pulmonary disease, *n* (%)[d] | 47 (22.6) | 20 (24.4) |
| Smoking, *n* (%) | – | – |
| Current | 49 (23.6) | 22 (26.8) |
| Former | 42 (20.2) | 21 (25.6) |
| Never | 117 (56.3) | 39 (47.6) |

Abbreviations: BMI, body mass index; IQR, interquartile range; VTE, venous thromboembolism.
[a]Data missing for one patient.
[b]With or without deep vein thrombosis.
[c]Some patients had more than one risk factor; patients with cancer, patients with pregnancy, and patients in the postpartum period were excluded.
[d]Including coronary artery disease, chronic heart failure, arrhythmia, peripheral artery disease, cerebrovascular disease, and chronic pulmonary disease.

 © 2025. The Author(s).

**Supplementary Table S3** Absolute and relative frequencies of patients within post-VTE functional status (PVFS) scale categories according to high (≥50th percentile) and low (<50th percentile) biomarker levels measured at study inclusion

| | PVFS scale grade | | | | |
|---|---|---|---|---|---|
| | 0 | 1 | 2 | 3 | 4 |
| D-dimer | | | | | |
| ≥ 50th percentile | 35 (25.7) | 40 (29.4) | 31 (22.8) | 20 (14.7) | 10 (7.4) |
| < 50th percentile | 56 (41.8) | 32 (23.9) | 30 (22.4) | 15 (11.2) | 1 (0.7) |
| Fibrinogen | | | | | |
| ≥ 50th percentile | 37 (26.8) | 35 (25.4) | 37 (16.8) | 23 (10.2) | 6 (4.3) |
| < 50th percentile | 56 (40.9) | 38 (27.7) | 23 (16.8) | 14 (10.2) | 6 (4.4) |
| FVIII | | | | | |
| ≥ 50th percentile | 28 (25.9) | 29 (26.9) | 27 (25.0) | 17 (15.7) | 7 (6.5) |
| < 50th percentile | 40 (37.4) | 32 (29.9) | 23 (21.5) | 10 (9.3) | 2 (1.9) |
| VWF | | | | | |
| ≥ 50th percentile | 31 (27.2) | 28 (24.6) | 31 (27.2) | 16 (14.0) | 8 (7.0) |
| < 50th percentile | 43 (37.7) | 34 (29.8) | 23 (20.2) | 13 (11.4) | 1 (0.9) |
| CRP | | | | | |
| ≥ 50th percentile | 35 (25.4) | 38 (27.5) | 38 (27.5) | 18 (13.0) | 9 (6.5) |
| < 50th percentile | 57 (41.6) | 35 (25.5) | 23 (16.8) | 19 (13.9) | 3 (2.2) |
| Troponin T | | | | | |
| ≥ 50th percentile | 17 (29.3) | 13 (22.4) | 13 (22.4) | 14 (24.1) | 1 (1.7) |
| < 50th percentile | 17 (32.7) | 13 (25.0) | 13 (25.0) | 8 (15.4) | 1 (1.9) |
| proBNP | | | | | |
| ≥ 50th percentile | 13 (23.2) | 15 (26.8) | 15 (26.8) | 11 (19.6) | 2 (3.6) |
| < 50th percentile | 20 (36.4) | 14 (25.5) | 11 (20.0) | 10 (18.2) | 0 (0.0) |

Abbreviations: CRP, C-reactive protein; FVIII, factor VIII; proBNP, N-terminal pro-B-type natriuretic peptide; PVFS, post-VTE functional status; VWF, von Willebrand factor antigen.
The relative frequencies relate to the absolute number of patients within high or low biomarker level categories. Troponin T and proBNP were only measured in patients with pulmonary embolism.

**Supplementary Table S4** AUC-ROC of biomarkers measured at study inclusion for discriminating patients with a post-VTE functional status scale grade >1 at 3 months

| | AUC-ROC (95% CI) |
|---|---|
| D-dimer | 0.58 (0.51–0.65) |
| Fibrinogen | 0.59 (0.52–0.66) |
| FVIII | 0.59 (0.51–0.67) |
| VWF | 0.62 (0.55–0.69) |
| CRP | 0.57 (0.51–0.64) |
| Troponin T[a] | 0.54 (0.42–0.65) |
| proBNP[a] | 0.56 (0.45–0.66) |

Abbreviations: AUC-ROC, area under the receiver operating characteristic curve; CRP, C-reactive protein; FVIII, factor VIII; proBNP, N-terminal pro-B-type natriuretic peptide; VTE, venous thromboembolism; VWF, von Willebrand factor antigen.
[a]Troponin T and proBNP were only evaluated in patients with pulmonary embolism.

 © 2025. The Author(s).

**Supplementary Table S5** Patient demographics and clinical characteristics of patients with and without missing biomarker values measured at 3 months

| | No missing biomarker values ($n = 245$) | Missing biomarker values ($n = 45$) |
|---|---|---|
| Female, *n* (%) | 97 (39.6) | 23 (51.1) |
| Age, median (IQR) | 55.3 (43.1–65.2) | 53.8 (42.9–60.0) |
| BMI, median (IQR)[a] | 27.7 (24.7–31.6) | 27.0 (22.8–31.6) |
| Type of VTE, *n* (%) | – | – |
| Pulmonary embolism[b] | 116 (47.3) | 18 (40.0) |
| Deep vein thrombosis | 129 (52.7) | 27 (60.0) |
| Unprovoked VTE, *n* (%) | 146 (59.6) | 28 (62.2) |
| Provoked VTE, *n* (%)[c] | 99 (40.4) | 17 (37.8) |
| Major persisting risk factor | 21 (8.6) | 4 (8.9) |
| Major transient risk factor | 33 (13.5) | 5 (11.1) |
| Minor transient risk factor | 56 (22.9) | 10 (22.2) |
| History of VTE, *n* (%) | 69 (28.2) | 16 (35.6) |
| History of cardiovascular or pulmonary disease, *n* (%)[d] | 61 (24.9) | 6 (13.3) |
| Smoking, *n* (%) | – | – |
| Current | 60 (24.5) | 11 (24.4) |
| Former | 56 (22.9) | 7 (15.6) |
| Never | 129 (52.7) | 27 (60.0) |

Abbreviations: BMI, body mass index; IQR, interquartile range; VTE, venous thromboembolism.
[a]Data missing for one patient.
[b]With or without deep vein thrombosis.
[c]Some patients had more than one risk factor; patients with cancer, patients with pregnancy, and patients in the postpartum period were excluded.
[d]Including coronary artery disease, chronic heart failure, arrhythmia, peripheral artery disease, cerebrovascular disease, and chronic pulmonary disease.

**Supplementary Table S6** Absolute and relative frequencies of patients within post-VTE functional status (PVFS) scale categories according to high (≥50th percentile) and low (<50th percentile) biomarker levels measured at 3 months

| | PVFS scale grade | | | | |
|---|---|---|---|---|---|
| | 0 | 1 | 2 | 3 | 4 |
| D-dimer | | | | | |
| ≥50th percentile | 44 (32.1) | 28 (20.4) | 38 (27.7) | 20 (14.6) | 7 (5.1) |
| <50th percentile | 40 (33.3) | 41 (34.2) | 23 (19.2) | 15 (12.5) | 1 (0.8) |
| Fibrinogen | | | | | |
| ≥50th percentile | 42 (31.8) | 28 (21.2) | 31 (23.5) | 22 (16.7) | 9 (6.8) |
| <50th percentile | 44 (33.8) | 42 (32.3) | 31 (23.8) | 13 (10.0) | 0 (0.0) |
| FVIII | | | | | |
| ≥50th percentile | 37 (28.2) | 35 (26.7) | 32 (24.4) | 19 (14.5) | 8 (6.1) |
| <50th percentile | 43 (35.8) | 33 (27.5) | 28 (23.3) | 16 (13.3) | 0 (0.0) |
| VWF | | | | | |
| ≥50th percentile | 38 (29.5) | 35 (27.1) | 31 (24.0) | 19 (14.7) | 6 (4.7) |
| <50th percentile | 46 (25.9) | 34 (26.6) | 30 (23.4) | 16 (12.5) | 2 (1.6) |
| CRP | | | | | |
| ≥50th percentile | 41 (29.5) | 38 (27.3) | 34 (24.5) | 17 (12.2) | 9 (6.5) |
| <50th percentile | 44 (36.1) | 32 (26.2) | 27 (22.1) | 18 (14.8) | 1 (0.8) |

 © 2025. The Author(s).

**Supplementary Table S6** (*Continued*)

| | PVFS scale grade | | | | |
|---|---|---|---|---|---|
| | 0 | 1 | 2 | 3 | 4 |
| Troponin T | | | | | |
| ≥ 50th percentile | 22 (39.3) | 10 (17.9) | 13 (23.2) | 10 (17.9) | 1 (1.8) |
| < 50th percentile | 9 (20.9) | 14 (32.6) | 14 (32.6) | 5 (11.6) | 1 (2.3) |
| proBNP | | | | | |
| ≥ 50th percentile | 16 (30.8) | 13 (25.0) | 14 (26.9) | 6 (11.5) | 3 (5.8) |
| < 50th percentile | 16 (30.8) | 13 (25.0) | 14 (26.9) | 9 (17.3) | 0 (0.0) |

Abbreviations: CRP, C-reactive protein; FVIII, factor VIII; proBNP, N-terminal pro-B-type natriuretic peptide; PVFS, post-VTE functional status; VWF, von Willebrand factor antigen.
The relative frequencies relate to the absolute number of patients within high or low biomarker level categories. Troponin T and proBNP were only measured in patients with pulmonary embolism.

**Supplementary Table S7** AUC-ROC and 95% confidence interval of biomarkers at 3 months for discriminating patients with a post-VTE functional status scale grade >1 at 3 months

| | AUC-ROC (95% CI) |
|---|---|
| D-dimer | 0.62 (0.55–0.69) |
| Fibrinogen | 0.63 (0.55–0.69) |
| FVIII | 0.57 (0.49–0.64) |
| VWF | 0.55 (0.48–0.63) |
| CRP | 0.58 (0.50–9.64) |
| Troponin T[a] | 0.53 (0.41–0.64) |
| proBNP[a] | 0.55 (0.43–0.67) |

Abbreviations: AUC-ROC, area under the receiver operating characteristic curve; CRP, C-reactive protein; FVIII, factor VIII; proBNP, N-terminal pro-B-type natriuretic peptide; VTE, venous thromboembolism; VWF, von Willebrand factor antigen.
[a]Troponin T and proBNP were only evaluated in patients with pulmonary embolism.

 © 2025. The Author(s).

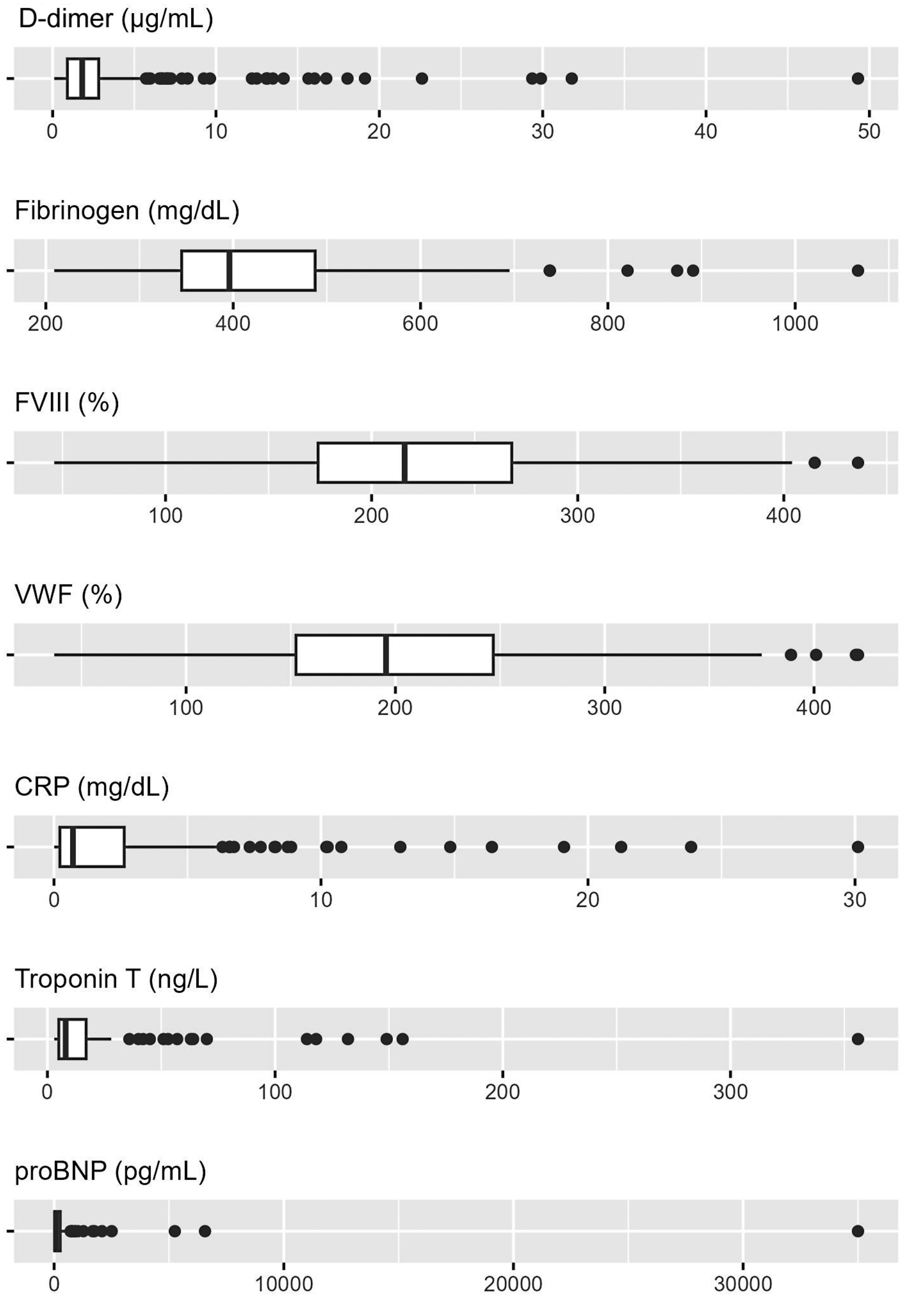


**Supplementary Fig. S1** Median (IQR) biomarker levels at study inclusion. Troponin T and proBNP were only measured in patients with PE. CRP, C-reactive protein; FVIII, factor VIII; IQR, interquartile range; PE, pulmonary embolism; proBNP, N-terminal pro-B-type natriuretic peptide; VWF, von Willebrand factor antigen.

 © 2025. The Author(s).

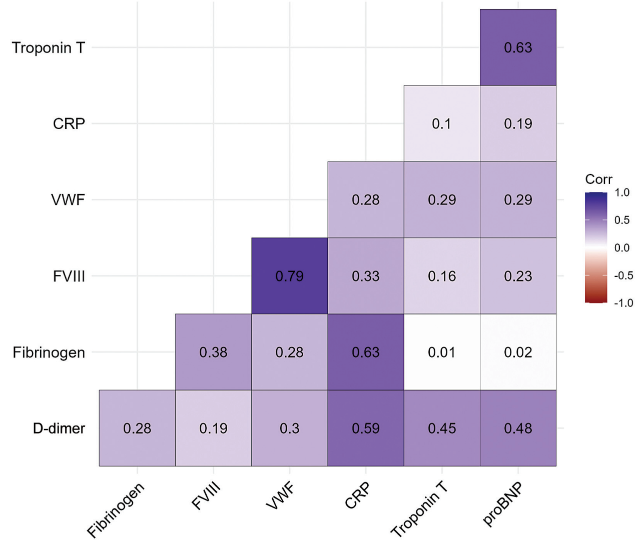


**Supplementary Fig. S2** Correlation matrix between all considered biomarkers measured at study inclusion. Spearman's rank correlation coefficients between all considered biomarkers are displayed in text within each box and graphically, ranging from −1 (dark red) over 0 (white) to 1 (dark blue). Troponin T and proBNP were only measured in patients with PE, therefore, their respective correlation coefficients only refer to this subgroup of patients. CRP, C-reactive protein; FVIII, factor VIII; proBNP, N-terminal pro-B-type natriuretic peptide; VWF, von Willebrand factor antigen.

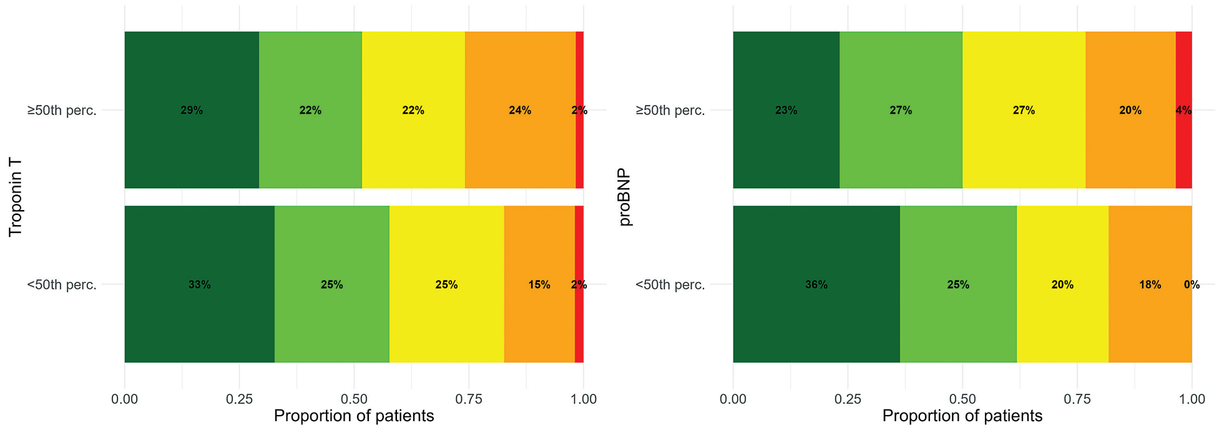


**Supplementary Fig. S3** Proportions of pulmonary embolism patients per post-VTE functional status (PVFS) scale category at 3 months with high (≥50th percentile) and low (<50th percentile) biomarker levels measured at study inclusion. Dark green refers to PVFS scale of 0 (no functional limitations), light green to 1 (negligible functional limitations), yellow to 2 (slight functional limitations), orange to 3 (moderate functional limitations), and red to 4 (severe functional limitations). perc, percentile; proBNP, N-terminal pro-B-type natriuretic peptide; VTE, venous thromboembolism.

 © 2025. The Author(s).

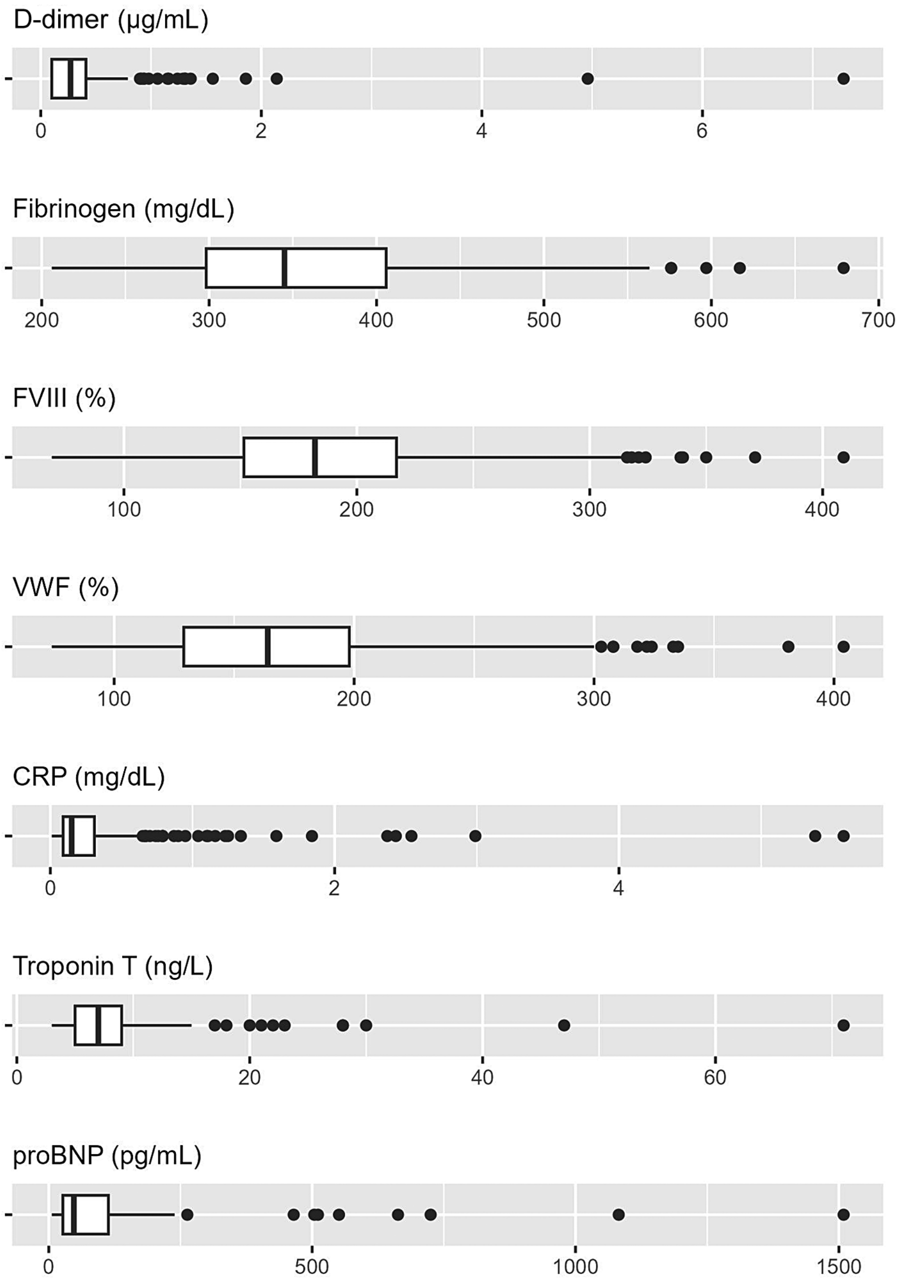


**Supplementary Fig. S4** Median (IQR) biomarker levels at 3 months. Troponin T and proBNP were only measured in patients with PE. CRP, C-reactive protein; FVIII, factor VIII; IQR, interquartile range; PE, pulmonary embolism; proBNP, N-terminal pro-B-type natriuretic peptide; VWF, von Willebrand factor antigen.

 © 2025. The Author(s).

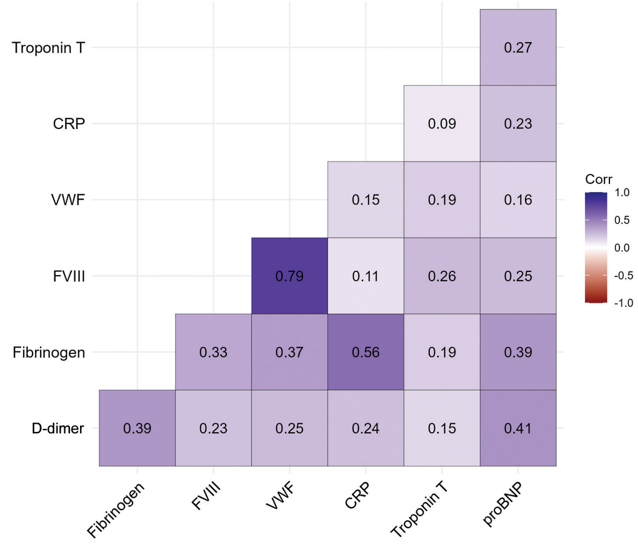


**Supplementary Fig. S5** Correlation between all considered biomarkers measured at 3 months. Spearman's rank correlation coefficients between all considered biomarkers are displayed in text within each box and graphically, ranging from −1 (dark red) over 0 (white) to 1 (dark blue). Troponin T and proBNP were only measured in patients with PE, therefore, their respective correlation coefficients only refer to this subgroup of patients. CRP, C-reactive protein; FVIII, factor VIII; PE, pulmonary embolism; proBNP, N-terminal pro-B-type natriuretic peptide; VWF, von Willebrand factor antigen.

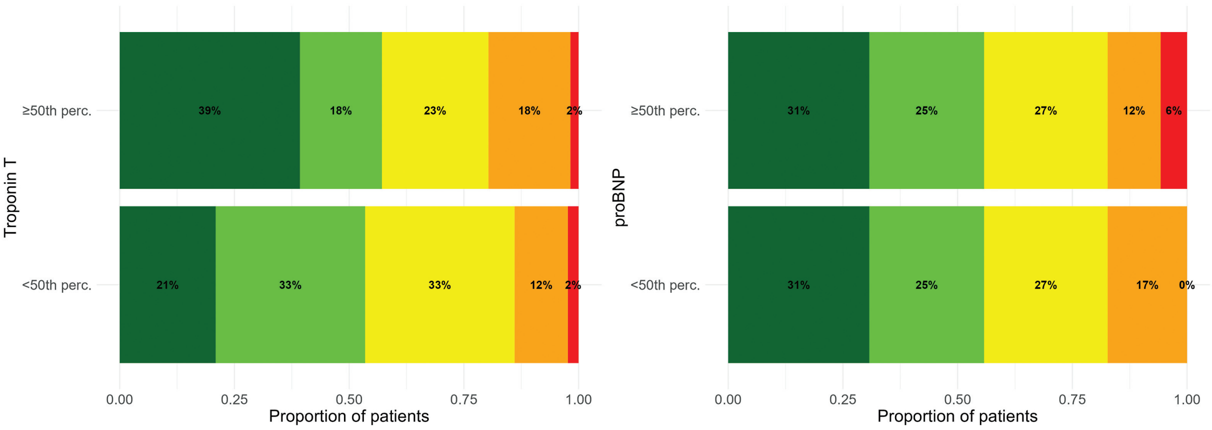


**Supplementary Fig. S6** Proportions of pulmonary embolism patients per post-VTE functional status (PVFS) scale category at three months with high (≥50th percentile) and low (<50th percentile) biomarker levels measured at 3 months. Dark green refers to PVFS scale of 0 (no functional limitations), light green to 1 (negligible functional limitations), yellow to 2 (slight functional limitations), orange to 3 (moderate functional limitations), and red to 4 (severe functional limitations). perc, percentile; proBNP, N-terminal pro-B-type natriuretic peptide; VTE, venous thromboembolism.

 © 2025. The Author(s).
